# Quercetin Reduces Inflammation and Protects Gut Microbiota in Broilers

**DOI:** 10.3390/molecules27103269

**Published:** 2022-05-19

**Authors:** Lei Sun, Lewei Guo, Gaoqing Xu, Zhiqiang Li, Michael Osei Appiah, Lianyu Yang, Wenfa Lu

**Affiliations:** College of Animal Science and Technology, Jilin Agricultural University, Changchun 130118, China; jzyxysl@163.com (L.S.); guolewei163@163.com (L.G.); xugaoqing1995@163.com (G.X.); lizhiqiangsky@126.com (Z.L.); moa4short@outlook.com (M.O.A.)

**Keywords:** LPS, quercetin, inflammatory response, intestinal microflora, broiler chicken

## Abstract

The aim of this study was to investigate the effects of quercetin on inflammatory response and intestinal microflora in broiler chicken jejuna. A total of 120 broiler chickens were allocated into 3 groups: saline-challenged broilers fed a basal diet (CTR group), lipopolysaccharide (LPS)-challenged broilers fed a basal diet (L group) and LPS-challenged broilers fed a basal diet supplemented with 200 mg/kg quercetin (LQ group). Our results showed that LPS significantly increased expression of tumor necrosis factor (TNF)-α, interleukin (IL)-1β, IL-6, IL-8, interferon (IFN)-γ, toll-like receptor (TLR)-4, *Bax, Caspase-3* and diamine oxidase activity (DAO), and decreased expression of zona occludens-1 *(*ZO-1*)*, Occludin and Bcl-2 in the jejunum, while dietary quercetin prevented the adverse effects of LPS injection. LPS injection significantly decreased the number of *Actinobacteria*, *Armatimonadetes* and *Fibrobacteriae* at the phylum level when compared to the CTR group. Additionally, at genus level, compared with the CTR group, the abundance of *Halomonas*, *Micromonospora*, *Nitriliruptor*, *Peptococcus*, *Rubellimicrobium*, *Rubrobacter* and *Slaclda* in L group was significantly decreased, while dietary quercetin restored the numbers of these bacteria. In conclusion, our results demonstrated that dietary quercetin could alleviate inflammatory responses of broiler chickens accompanied by modulating jejunum microflora.

## 1. Introduction

Chicken enteritis is a comprehensive intestinal disease caused by bacteria, viruses, parasites and a variety of management factors. In production, the incidence rate of enteritis is high; although the mortality rate is low, the direct economic loss is huge [1,2,3]. In recent decades, antibiotics have been widely used to prevent or control bacterial infections in animals. However, overuse of antibiotics leads to multiple drug resistance (MDR) and has affected the health of animals [4]. Thus, dietary supplementation of plant-derived compounds with anti-inflammatory and antioxidant effects has been studied as a substitute for antibiotics. Some flavonoids, such as naringin and luteolin, have been found to relieve intestinal inflammation [5,6].

Quercetin is a natural flavonoid. Its chemical name is 3,3′,4′,5,7-pentahydroxy-flavone, and it has antioxidant, antitumor, anti-inflammatory, free radical scavenging and other biological actions. Meanwhile, This study proposed that quercetin could play a beneficial role in intestinal barrier function [7]. Quercetin inhibited the expression of TNF-α, IL-1β, IL-6 and GM-CSF in macrophages induced by lipopolysaccharides (LPS) [8]. The effects of flavonoids on body health depends largely on the transformation of intestinal microflora [9]. Studies have found that quercetin could reverse diet-induced changes in the composition of intestinal microorganisms related to obesity and could effectively reduce the induction effects of a high-fat sucrose diet [10]. It also had an anti-inflammatory effect, suppressing the expression of cell adhesion molecules in LPS-induced rat intestinal microvascular endothelial cells [11]. The study found that dysbiosis was a causal or contributing factor in the pathogenesis of inflammatory bowel disease [12]. The use of high-throughput sequencing of 16s rRNA genes revealed that intestinal inflammation was generally accompanied by changes in the composition of the intestinal microbiome (dysbiosis). Meanwhile, intestinal microflora dysbiosis also led to gastrointestinal and inflammatory disorders [13,14]. Maternal fermented diet could ameliorate neonatal colonic inflammation by inducing an abundance of gut *Lactobacillus reuteri* [15]. Licochalcone A alleviated ulcerative colitis by maintaining tight junction protein expression (maintaining integrity of the intestinal barrier), inhibiting inflammation levels and regulating intestinal barrier-associated bacteria [16]. In our present study, we studied the influence of quercetin on intestinal inflammation and then analyzed the effects of quercetin on intestinal microflora of broiler chicken jejuna challenged with LPS.

## 2. Results

### 2.1. Effect of Dietary Quercetin on the Intestinal Inflammation in Broiler Chickens Challenged with LPS

**Figure 1:**
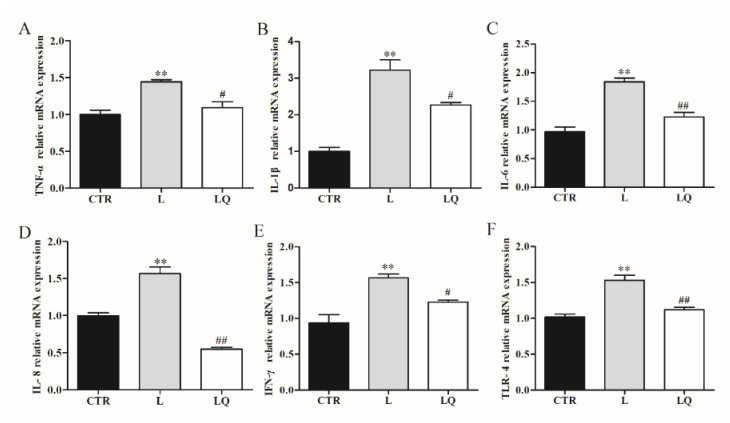
The mRNA levels of inflammatory cytokines and toll-like receptor (TLR)-4 in jejunum. Tumor necrosis factor (TNF)-α (**A**), interleukin (IL)-1β (**B**), IL-6 (**C**), IL-8 (**D**), interferon (IFN)-γ (**E**) and TLR-4 (**F**) mRNA expressions are determined using RT-PCR. Values in bar graphs are means ± standard error of the mean (SEM) of at least 3 independent experiments performed. Data are analyzed using one-way analysis of variance (ANOVA) accompanied by Tukey’s post-hoc test. ** *p* < 0.01 vs. CTR and ## *p* < 0.01 or # *p* < 0.05 vs. L. CTR (control group), L (LPS group) and LQ (quercetin group).

**Figure 2:**
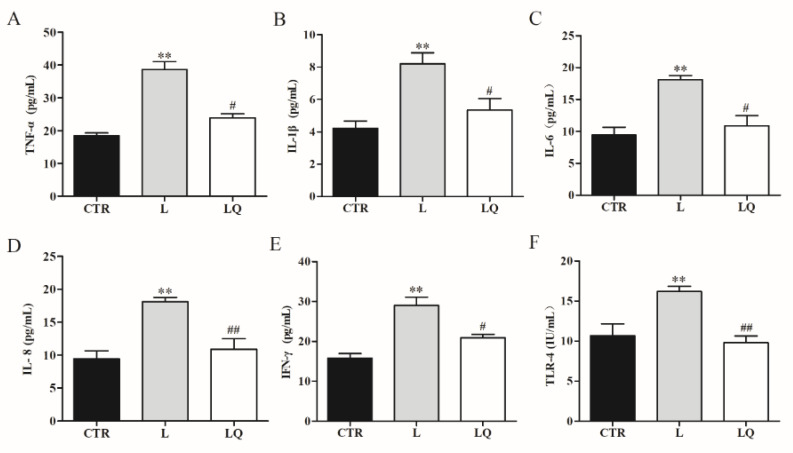
Protein content of inflammatory cytokines and toll-like receptor (TLR)-4 in jejunum. Tumor necrosis factor (TNF)-α (**A**), interleukin (IL)-1β (**B**), IL-6 (**C**), IL-8 (**D**), interferon (IFN)-γ (**E**) and TLR-4 (**F**) are determined using ELISA. Values in bar graphs are means ± standard error of the mean (SEM) of at least 3 independent experiments performed. Data are analyzed using one-way analysis of variance (ANOVA) accompanied by Tukey’s post-hoc test. ** *p* < 0.01 vs. CTR and ## *p* < 0.01 or # *p* < 0.05 vs. L. CTR (control group), L (LPS group) and LQ (quercetin group).

To investigate the regulatory effects of quercetin on the intestinal inflammation of broiler chickens, mRNA expressions and protein content of toll-like receptor (TLR)-4 and inflammatory cytokines tumor necrosis factor (TNF)-α, interleukin (IL)-1β, IL-6, IL-8 and interferon (IFN)-γ were detected using RT-PCR and ELISA. As shown in Figure 1 and Figure 2, LPS challenge significantly increased mRNA expression and protein content of TNF-α, IL-1β, IL-6, IL-8, IFN-γ and TLR-4 in the jejuna (*p* < 0.01), while dietary quercetin decreased the expression of these genes induced by LPS.

### 2.2. Effect of Dietary Quercetin on the Intestinal Barrier of Broiler Chickens Challenged with LPS

**Figure 3:**
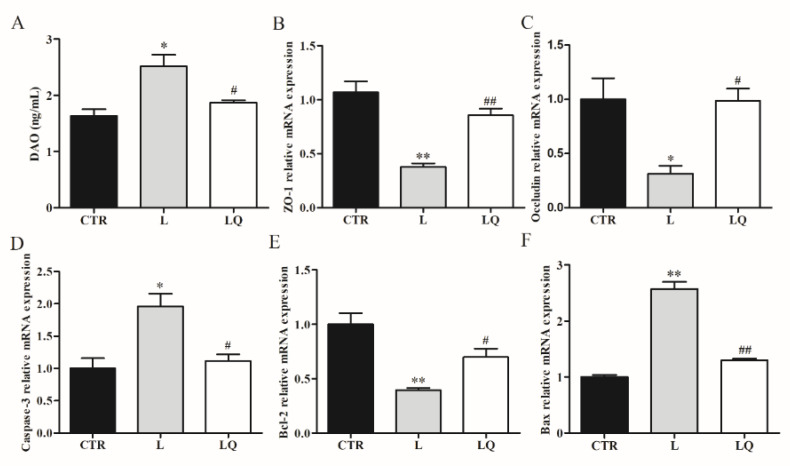
Effects of dietary quercetin on expression of apoptosis-related genes, tight junction protein genes and DAO in broiler chicken jejuna. The relative mRNA expression levels of apoptosis-related genes, including Caspase-3 (**A**), Bcl-2 (**B**) and Bax (**C**) are detected and analyzed (*n* = 3). The relative mRNA expression levels of tight junction-related genes, including zona occludens-1 (ZO-1) (**D**) and Occludin (**E**) are detected and analyzed (*n* = 3). The diamine oxidase activity (DAO) (**F**) level is detected and analyzed (*n* = 3). Data are analyzed using one-way ANOVA accompanied by Tukey’s post-hoc test. ** *p* < 0.01 or * *p* < 0.05 vs. CTR and ## *p* < 0.01 or # *p* < 0.05 vs. L. CTR. CTR (control group), L (LPS group) and LQ (quercetin group).

We further investigated the effects of quercetin on the intestinal barrier and examined the expression of apoptosis-related genes, tight junction protein genes and DAO in broiler chickens. As shown in Figure 3, LPS challenge significantly increased the mRNA expression of pro-apoptosis genes *Caspase-3* and *Bax* (*p* < 0.01), while it decreased the mRNA expression of anti-apoptosis gene *Bcl-2* (*p* < 0.01), tight junction protein genes *zona occludens-1* (ZO-1) and *Occludin* (*p* < 0.05) when compared to the CTR group. The altered mRNA expressions of these apoptosis-related genes and tight junction protein genes were reversed with dietary quercetin. We detected the diamine oxidase activity (DAO) level in plasma and found that dietary quercetin significantly relieved the increased DAO (*p* < 0.05) level in plasma induced by LPS.

### 2.3. 16S rRNA Sequencing and Total out Analysis

**Figure 4:**
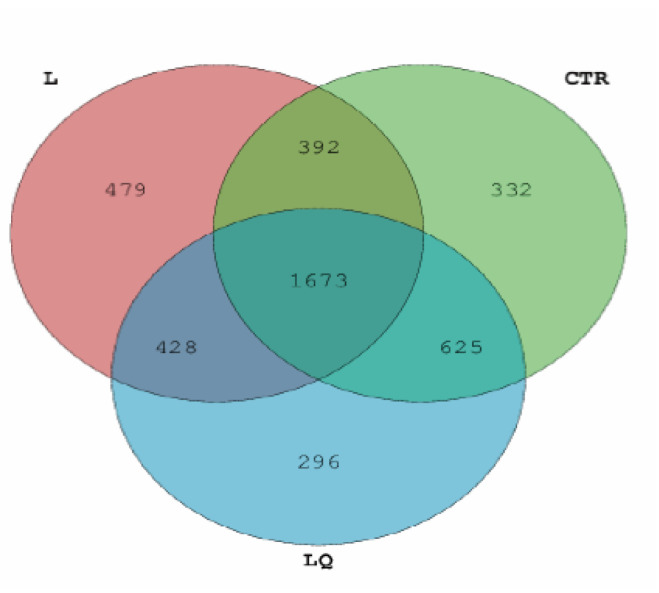
Total operational taxonomic unit (OTU) analyses. Each circle represents a group; the green is the CTR group, the pink is the L group, and the blue is the LQ group. The overlapping areas between circles indicate the number of shared OTUs between groups, and the number of shared or unique OTUs of groups contained within each block. CTR (control group), L (LPS group) and LQ (quercetin group).

Total DNA extracted from 27 jejunum samples were collected from three groups (*n* = 9). There were 297,258, 279,105 and 27,447 sequences of intestinal microbiota found in CTR, L and LQ, respectively. The proportions for shared and unique OTUs in the three groups are intuitively indicated in the Venn diagram in Figure 4. There were 1673 shared OTUs contained in CTR, L and LQ groups; the unique OTUs were 332, 479 and 296, respectively (Figure 4).

### 2.4. Diversity and Similarity of Microbial Community

The diversity of microbial communities among CTR, L and LQ groups was measured based on the alpha-diversity indices, such as chao1, Simpson and Shannon index (Table 1). There were no significant differences among the three groups, indicating that LPS and quercetin caused no changes in microbial community diversity.

**Figure 5:**
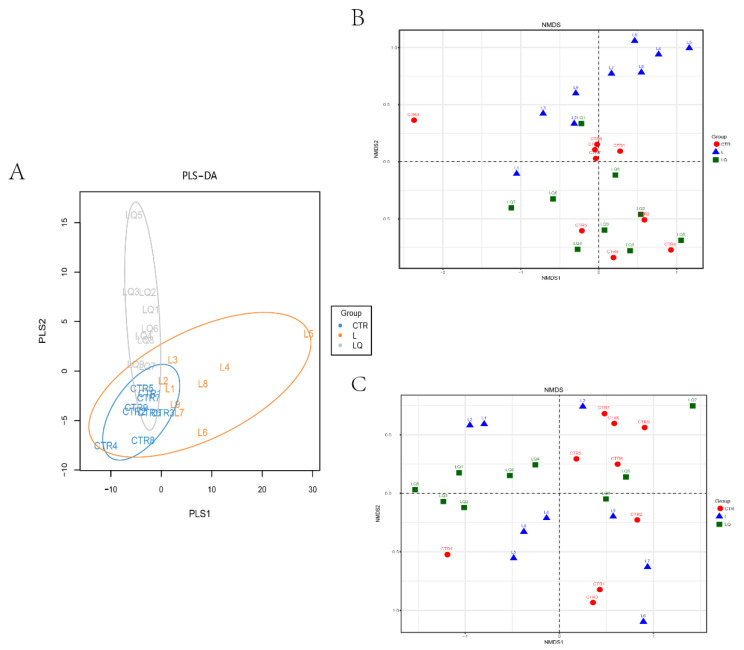
Similar analyses of microbial community. (**A**) Partial least squares discriminant analysis (PLS-DA) diagram. Each point represents a sample; the points with the same color belong to the same group, and the points of the same group are marked by ellipses. Nonmetric multidimensional Scaling (NMDS) analyses are based on the UniFrac distance. The UniFrac results are divided into unweighted Unifmc (**B**) and weighted UniFrac (**C**). Each point represents a sample, and the points with different colors belong to different groups. CTR (control group), L (LPS group) and LQ (quercetin group).

Partial least squares discriminant analysis (PLS-DA) depicted the differences between and distance of samples by analyzing the different groups of the OTU. It applied variance decomposition to indicate the differences between two-fold samples on a two-dimensional coordinate graph. The circles of the different treatment groups in the PLS-DA map have overlapping regions, indicating that the differences between the groups were not very high (Figure 5A).

Beta-diversity analysis was used to investigate the similarity of community structure among the three groups and nonmetric multidimensional scaling (NMDS) analysis was performed for unweighted Unifmc and weighted UniFrac (Figure 5B,C) distance matrix by R software. The results of unweighted Unifmc (Figure 5B) showed that there was a grouping pattern between L and other groups (CTR and LQ) and a higher similarity between the CTR and LQ samples. However, the results of weighted UniFrac (Figure 5C) showed that there was no grouping pattern between groups.

### 2.5. Phylum Level Abundance and Significance Difference of the Three Groups

**Figure 6:**
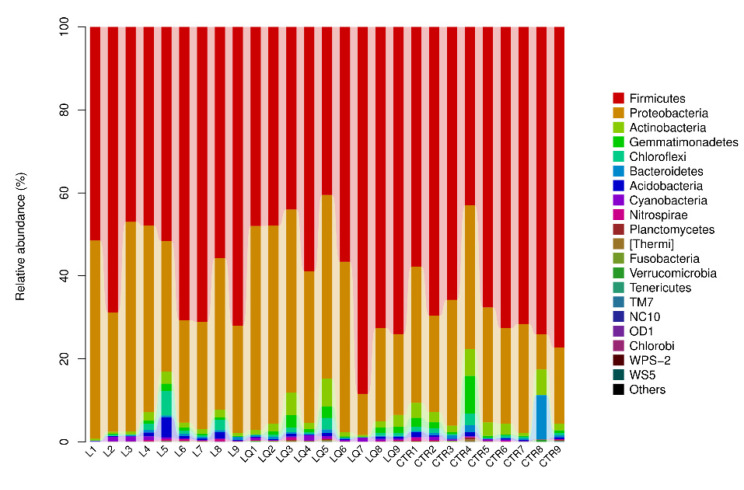
Phylum level community composition and abundance distribution map. The horizontal coordinate is arranged according to the sample name, and each bar graph represents a sample. Colors are used to distinguish each taxon. The vertical coordinates represent the relative abundance of each taxon. In these samples, the longer the column, the higher relative abundance of the taxon. CTR (control group), L (LPS group) and LQ (quercetin group).

**Figure 7:**
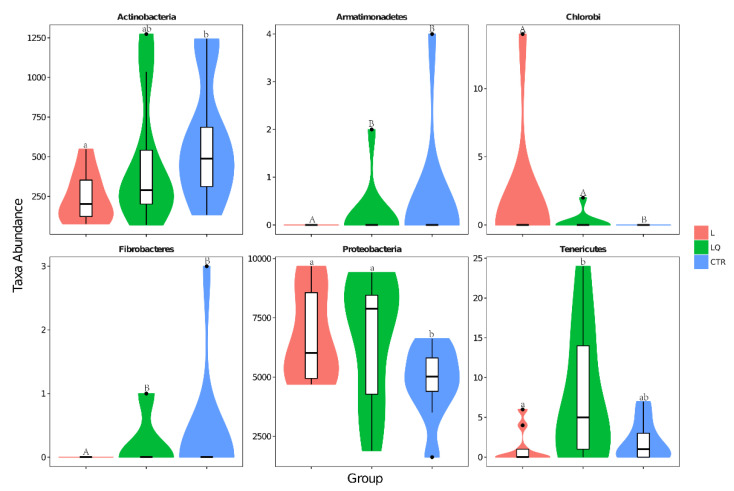
Classifications of bacteria show significant differences at the phylum level. The border of boxplot represents the upper and lower quartile spacing, the horizontal line represents the median value and the upper and lower whisker represents 1.5 times the IQR range outside the upper and lower quartile, respectively. The symbol • indicates the extreme value beyond the range. a, b indicate significant differences at *p* < 0.05; A, B indicate significant differences at *p* < 0.01. The same letters indicate no significant differences. CTR (control group), L (LPS group) and LQ (quercetin group).

As shown in Figure 6, *Firmicutes* were the most dominant bacteria at the phylum level, accounting for 66.60%, 59.59% and 58.99% in the CTR, L and LQ groups, respectively, followed by *Proteobacteria*, accounting for 24.96%, 35.19% and 34.99% in the CTR, L and LQ groups respectively. Another important bacterium with relatively low proportions was *Actinobacteria*, which had 3.17%, 1.26% and 2.47%, respectively. Results of Phylum level abundance in different groups are shown in Figure 7. The results showed that, at the phylum level, intraperitoneal injection of LPS could decrease the number of *Actinobacteria* (*p* < 0.05), *Armatimonadetes* (*p* < 0.01) and *Fibrobacteriae* (*p* < 0.01) when compared to the CTR group in jejuna of broiler chickens, while dietary quercetin could restore the number of these bacteria. *Chlorobi* (*p* < 0.01) and *Proteobacteria* (*p* < 0.05) in L group and LQ group were significantly abundant than in the CTR group.

### 2.6. Genus Level Abundance and Significance Difference of the Three Groups

**Figure 8:**
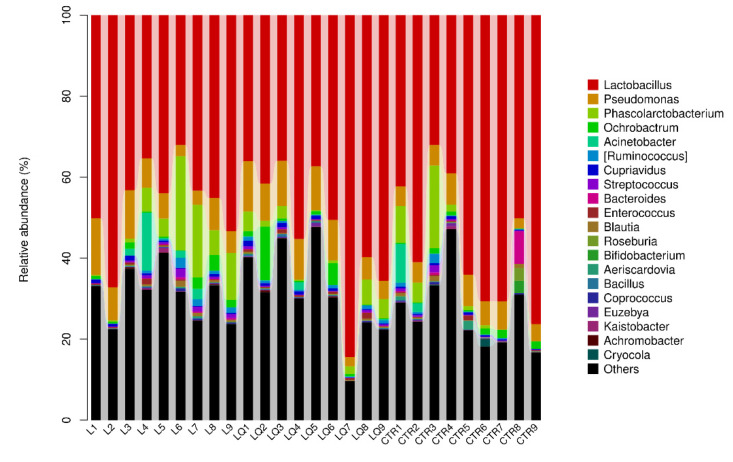
Genus-level community composition and abundance distribution map. The horizontal coordinate is arranged according to the sample name, each bar graph represents a sample and colors are used to distinguish each taxon. The vertical coordinate represents the relative abundance of each taxon. In these samples, the longer the column, the higher the relative abundance of the taxon. CTR (control group), L (LPS group) and LQ (quercetin group).

**Figure 9:**
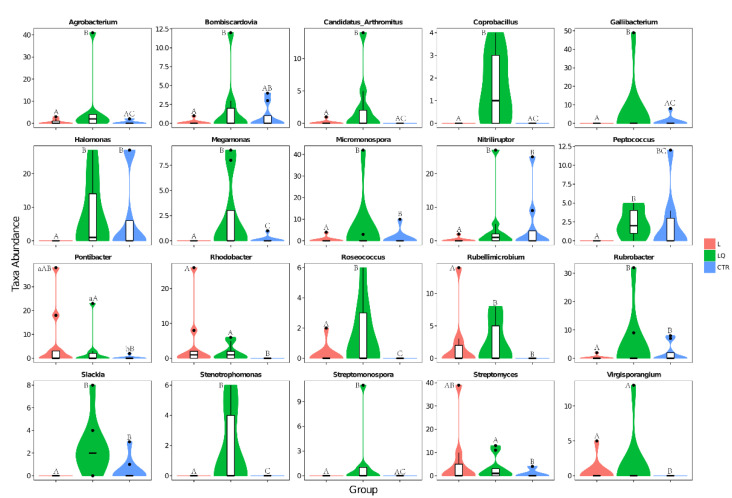
Classifications of bacteria show significant differences at the genus level. The border of the boxplot represents the upper and lower quartile spacing, the horizontal line represents the median value and the upper and lower whisker represents 1.5 times the IQR range outside the upper and lower quartile, respectively. The symbol • indicates the extreme value beyond the range. a, b indicate significant differences at *p* < 0.05; A, B and C indicate significant differences at *p* < 0.01. The same letters indicate no significant differences. CTR (control group), L (LPS group) and LQ (quercetin group).

The most abundant bacterium at the genus level was *Lactobacillus* and its abundance levels in the CTR, L and LQ groups were 56.24%, 45.98% and 51.87%, respectively. Unclassified *Moraxellaceae* also had 12.42%, 16.98% and 18.94% abundance in CTR, L and LQ groups, respectively. Another abundant genus was *Pseudomonas;* its abundance levels in the CTR, L and LQ groups were 5.56%, 7.48% and 8.46%, respectively (Figure 8). Results of genus-level abundance in different groups are shown in Figure 9. At the genus level, compared with the CTR group, the abundance of *Halomonas, Micromonospora, Nitriliruptor, Peptococcus, Rubellimicrobium, Rubrobacter* and *Slacldain* (*p* < 0.05) in L group were significantly decreased, while the LQ group restored the number of these bacteria. The number of *Agrobacterium, Candidatus_Arthromitus, Coprobacillus, Gallibacterium* and *Streptomonospora* (*p* < 0.01) in LQ group were significantly more abundant than in CTR and L groups. Compared with the CTR group, the abundance of *Rhodobacter, Virgisporangium* (*p* < 0.01) in L group increased significantly, while *Rhodobacter, Virgisporangium, Pontibacter* and *Streptomyces* (*p* < 0.01) in LQ group increased significantly.

## 3. Discussion

The gut is the largest immunity organ in an animal’s body, and the benefits to animals maintaining a healthy gut are immeasurable [17]. Previous studies have shown that quercetin has anti-inflammatory effects [18,19]. Supplementation of dietary quercetin could mitigate intestinal inflammatory damage, as well as promote intestinal structure and immune barrier integrity in aged breeder hens [20]. In addition, intestinal inflammation could also be alleviated by regulating intestinal flora [21] The present study demonstrated, for the first time, that dietary quercetin could alleviate inflammatory responses induced by LPS injection and modulate the jejunum microflora of broiler chickens.

Cytokines are endogenous mediators of the immune system and are responsible for controlling inflammatory responses [22]. The intestinal barrier regulates the passage of microorganisms, proinflammatory molecules, antigens and toxins [23], whereas external stimuli induce oxidative stress, which ultimately increases proinflammatory cytokines, leading to intestinal epithelial cell damage and apoptosis [24]. Dietary antioxidants reduce intestinal inflammation by inhibiting proinflammatory enzymes [25]. Dietary quercetin also reduced cellular apoptosis in Japanese quails [26]. Similarly, this study showed that LPS-challenged chickens exhibited increased expression of inflammatory cytokines (TNF-α, IL-1β, IL-6, IL-8 and IFN-γ), TLR-4 and apoptosis-related genes (Bax and Caspase3), and decreased the expression of Bcl-2 in jejuna of broiler chickens, while dietary quercetin reversed the expression of these genes induced by LPS. Tight junction proteins (ZO-1 and occludin) and DAO affect the normal function of the intestinal mucosal barrier [27]. We found that LPS injection decreased the mRNA expression of ZO-1 and occludin, increased the level of DAO, and caused damage to the structure and function of the jejunum barrier; dietary quercetin prevented the adverse effects of LPS injection. Previous studies supported this research. It has been demonstrated that LPS can induce intestinal inflammation and barrier dysfunction in broilers [28]. Quercetin could ameliorate the inflammatory response of human retinal pigment epithelial cells [29]. Compared to untreated mice with allergic airway inflammation, quercetin supplementation improved histopathological changes in lung and decreased levels of inflammatory cytokine and cell apoptosis [18]. Recently, studies also showed that dietary quercetin could counteract colitis in mice [30,31] Quercetin also protected intestinal porcine enterocyte cells from H_2_O_2_-induced apoptosis [32]. Quercetin could alleviate intestinal barrier disruption and inflammation in acute necrotizing pancreatitis of rats [33]. Supported by previous studies, the results showed that dietary quercetin could alleviate inflammatory response and barrier dysfunction in the jejuna of broilers induced by LPS.

Mounting evidence has shown that intestinal microflora affect immune function and inflammatory response [34]. Previous studies have shown that LPS treatment could lead to inflammatory response and microflora disorder [35]. Dietary quercetin could alleviate colitis severity in Citrobacter rodentium-infected mice by modulating gut microflora [30]. Consistently, our results showed that LPS injection could significantly decrease the number of *Actinobacteria*, *Armatimonadetes* and *Fibrobacteriae* at the phylum level. Additionally, at genus level, compared with the CTR group, the abundance of *Halomonas*, *Micromonospora*, *Nitriliruptor*, *Peptococcus*, *Rubellimicrobium* and *Rubrobacter* in L group were significantly decreased; dietary quercetin could restore the numbers of these bacteria. Dietary quercetin also significantly increased the number of *Rhodobacter*, *Virgisporangium*, *Pontibacter* and *Streptomyces*. These results indicated that dietary quercetin could at least partly restore microbial communities in jejuna of broilers caused by LPS and increase some beneficial bacteria. The quantitative changes in some specific bacteria in the present study were supported by previous studies. Studies have pointed out that the abundance levels of *Halomonas*, *Peptococcus*, and *Rubrobacter* can be associated with intestinal inflammation, and are increased when intestinal inflammation occurs [36,37,38]. *Streptomyces* and *Micromonospora*, as members of *Actinobacteria*, have been shown to have anti-inflammatory and antioxidant effects [39,40,41]. It has been shown that *Rhodobacter* spheroids attenuate inflammation and directly regulate T cell response [42]. Quercetin has antibacterial effects and changes cecal microbial population and distribution in broilers [43]. In rats, quercetin could restore gut microbiota dysbiosis induced by a high-fat sucrose diet [44]. This research showed that quercetin could protect the gut from inflammatory responses by modulating microbial communities. 

However, there were some limitations in the present study. The effects of specific bacteria regulated by quercetin on the inflammatory responses of the gut were not investigated. Therefore, experiments in vivo investigating the role of some specific bacteria on gut inflammatory responses in broilers might help clarify the anti-inflammatory effects of dietary quercetin via microflora.

## 4. Conclusions

200 mg/kg quercetin improved LPS-induced inflammation and apoptosis in broilers, downregulating TNF-α, IL-1β, IL-6, IL-8, IFN-γ, TLR-4, Bax, Caspase-3 and DAO and upregulating the expression of ZO-1, Occludin and Bcl-2. Meanwhile, Quercetin also ameliorated LPS-induced changes in jejunal microbiota and improved gut immunity with increased abundance of *Peptococcus*, *Rubellimicrobium*, *Rubrobacter* and *Slaclda*. In conclusion, this research showed that dietary quercetin could alleviate inflammatory responses in broiler chickens accompanied by modulating jejunum microflora. This study will have certain reference significance for the application of quercetin to the development of broiler production.

## 5. Materials and Methods

Ethical approval for the present study was obtained from the Ethical Committee of the Jilin Agricultural University, China.

### 5.1. Animal Treatment and Sample Preparation

One hundred and twenty arbor acre broiler chickens (1-day-old) were kept for three weeks and their basal diet was formulated to meet NRC (1994) recommendations (Table 2). The broiler chickens were randomly allocated into 3 groups (*n* = 40): control group (CTR), LPS group (L) and quercetin group (LQ). Broiler chickens in the CTR and L groups were fed a basal diet, while broiler chickens in LQ groups were fed a basal diet containing 200 mg/kg of quercetin. The broiler chickens in the L and LQ groups were intraperitoneally injected with LPS on days 16, 18 and 20, respectively. The injection volume was 0.5 mg/kg (sigma company, serotype 055 B55, diluted with sterilized saline to 0.5 mg/mL). Additionally, broiler chickens in the CTR group were injected with the same volume of saline as LPS treatment. Feed and water were provided ad libitum. Light was applied 24 h per day. Broiler chickens were slaughtered at 21 days old. The jejuna were collected at about 10 cm distal to the duodenum, opened longitudinally, then flushed gently and repeatedly with phosphate-buffered saline (pH 7.4). They were then transferred into sterile frozen tubes and flash-frozen in liquid nitrogen. Segments of approximately 2 cm of mid-jejunum were rinsed with phosphate-buffered saline and kept in formalin solution (10%) for histological examination. At the same time, twelve broiler chickens from different groups were randomly selected and slaughtered by bleeding from the jugular vein. Their whole blood samples were collected using EDTA anticoagulant vacuum tubes. Plasma was then separated by centrifuging at 2000 rpm for 20 min and stored at −20 °C for further analysis.

### 5.2. Quantitative Real-Time Polymerase Chain Reaction (qRT-PCR) Analysis

Detection of mRNA expression of inflammatory cytokines, apoptosis related genes, tight junction protein genes and TLR-4 was carried out using qRT-PCR, and β-Actin was used as an interior control. Primers were synthesized by Shanghai Biotech (Table 3). Reverse transcription to cDNA was conducted using TaKaRa reverse transcription kit and qRT-PCR was also performed with TB Green Supermix (Takara, Tokyo, Japan) real-time PCR system in a 20 μL reaction mixture comprising 2 μL cDNA template, 10 μL SYBR Premix Ex TaqII, 0.4 µL ROX reference DyeII, 0.8 µL Forward and Reverse Primer and 6 μL nuclease-free water. The separate sample was analyzed into four repeats. The relative expression fraction of the target gene was calculated by means of the 2−ΔΔCt method and expressed in comparison with the β-Actin gene.

### 5.3. Enzyme-Linked Immunosorbent Assay (ELISA)

Jejunum tissue samples were treated with protease inhibitor and phosphate-buffered saline and then subjected to homogenization. Finally, the samples were centrifuged at 5000× *g* for 10 min, and the supernatant was used for ELISA. The contents of toll-like receptor (TLR)-4, inflammatory cytokines tumor necrosis factor (TNF)-α, interleukin (IL)-1β, IL-6, IL-8 and interferon (IFN)-γ were measured by ELISA using a commercial kit (Shanghai Enzyme-linked Biotechnology Co., Ltd., China), according to the manufacturer’s protocol.

### 5.4. Determination of Diamine Oxidase Activity (DAO) Level

After collection from different groups of broiler chickens, The DAO in the plasma was determined using a microplate reader, following the manufacturers’ instructions (Shanghai Langdun Biotech, Shanghai, China).

### 5.5. Extraction of Total DNA and Sequencing of 16S rRNA

Total DNA in the jejuna was extracted using the Fast DNA SPIN Extraction Kits (MP Biomedicals, Santa Ana, CA, USA) according to the manufacturer’s instructions. The purity of the DNA extracts was measured by 0.8% agarose gel electrophoresis. The PCR reaction procedure: 5 µL 5 × Q5 reaction buffer, 5 µL 5 × Q5 High-Fidelity GC buffer, 0.25 µL of Q5 High-Fidelity DNA Polymerase, 2 µL 2.5 mM dNTPs, 1 µL 10 µM Forward and Reverse Primer, 10 ng Template DNA and ddH2O were added to reach the final volume to 25 µL. PCR reaction conditions were as follows: 98 °C for 2 min with 25 cycles, 98 °C for 15 s, 55 °C for 30 s, 72 °C for 30 s and then a final extension at 72 °C for 5 min. The PCR product was separated and recovered by 2% agarose gel electrophoresis and quantified using Quant-iT PicoGreen dsDNA Assay Kits (Invitrogen, Carlsbad, CA, USA).

The DNA was sequenced using the Illumina MiSeq platform obtained from Shanghai Personal Biotechnology Co., Ltd. (Shanghai, China). All the raw sequences obtained from the Illumina MiSeq were filtered, and FLASH software (v1.2.7) was used to sort and evaluate the quality of the original data. The obtained sequences were clustered and ≥97% sequence identity was merged into the OTU [45]. The sequence with the highest abundance in each operational taxonomic unit (OTU) was then selected as the representative sequence of the OTU using QIIME software, and OTU classification was performed by BLAST search in the Greengenes database [46]. OTUs whose abundance values were less than 0.001% of the total sequencing volume of all samples [47] and rare OTUs were removed. According to the obtained OTU abundance matrix, R software was used to calculate the number of OTUs in the three groups, and a Venn chart was drawn. The QIIME software was used to calculate the α diversity analysis (Chao1, Simpson Mean and Shannon Mean) to determine the abundance and diversity of the sequences. Nonmetric multidimensional scaling (NMDS) analyses of Unweighted and Weighted UniFrac distance matrices were performed using R software to assess similarities in the structure and distribution of microbial genetic communities in the samples [48,49]. The phylum and genus intestinal flora composition and abundance distribution table were obtained using QIIME software.

### 5.6. Statistical Analysis

SPSS software 23.0 (IBM Corp, Armonk, NY, USA) was used for data analysis. The data were expressed as means ± standard error of the mean (SEM), analyzed for significance by one-way analysis of variance (ANOVA) accompanied by Tukey’s honestly significant difference post-hoc test. *p* < 0.05 meant that the difference was statistically significant.

## Figures and Tables

**Table 1:** The alpha-diversity indexes of data distribution.

Group *	Chaol	STD	Simpson	STD	Shannon	STD
	Mean		Mean		Mean	
L	1187.048	199.8716	0.950465	0.026915	6.39	0.682486
LQ	1287.091	181.8062	0.967144	0.010034	7.2	0.229627
CTR	1098.337	188.4453	0.930107	0.062601	6.26	0.773747

* L (LPS group), LQ (quercetin group), and CTR (control group).

**Table 2:** Formulation and nutrient compositions of basal diet.

Ingredient (%)	1–21 Days	Nutritional Index (g/kg)	1–21 Days
Corn	58.63	Crude protein	21
Soybean meal	32.07	Metabolism energy (MJ/kg)	3050
Soybean oil	3.53	Moisture	12.38
Corn gluten meal	2	Dry matter	87.62
Limestone powder	1.17	Crude fat	6.1
DL-methionine (98%)	0.23	Crude fiber	3.17
Lysine (98%)	0.38	Crude ash	5.55
L-threonine	0.05	Calcium	0.9
Choline chloride	0.1	Total phosphorus	0.64
Calcium hydrogen phosphate	1.03	Available phosphorus	0.41
Sodium chloride	0.33	Sodium	0.15
Broiler chickens vitamins *	0.2	Chlorine	0.23
Broiler chickens trace elements †	0.15	Total lysine	1.35
Complex enzyme	0.02	Total methionine	0.55
Phytase (10,000 units)	0.01	Total sulfur-containing amino acid	0.88
L-tryptophan (20%)	0.1	Total threonine	0.83
Total	100	Total tryptophan	0.25

* Vitamin for broiler chickens provided per kg of diet: VA, 10,000 IU; VB1, 2 mg; VB2, 7 mg; VB6, 4 mg; VB12, 0.02 mg; VD, 4000 IU; VE, 25 mg; VK, 2 mg; biotin, 0.1 mg; folic acid, 1.2 mg; niacinamide, 40 mg; calcium pantothenate, 10 mg. † Fe (from ferrous sulfate), 100 mg; Mn (from manganese sulfate), 100 mg; Zn (from zinc oxide), 65 mg; Cu (from copper sulfate), 10 mg; Se (from sodium selenite), 0.3 mg; I (from calcium iodate), 0.7 mg.

**Table 3:** Information on primers of real-time quantitative PCR.

Genes	Primer Sequence (5′–3′)	Genebank No.	Size (bp)
TNF-α	F: TGGGAAGGGAATGAACCCTC	NM_204267.1	118
	R: AACTGGGCGGTCATAGAACA		
IL-1β	F: AGCAGCCTCAGCGAAGAGACC	NM_204524.1	90
	R: GTCCACTGTGGTGTGCTCAGAATC		
IL-6	F: TTCACCGTGTGCGAGAACAGC	NM_204628.1	80
	R: CAGCCGTCCTCCTCCGTCAC		
IL-8	F: AGGATGGAAGAGAGGTGTGC	NM_205498.1	80
	R: CTGAGCCTTGGCCATAAGTG		
TLR4	F: AGGCACCTGAGCTTTTCCTC	NM_001030693.1	96
	R: TACCAACGTGAGGTTGAGCC		
IFN-γ	F: CACTGACAAGTCAAAGCCGC	NM_205149.1	87
	R: ACCTTCTTCACGCCATCAGG		
BCL-2	F: GAGTTCGGCGGCGTGATGTG	NM_205339.2	92
	R: TTCAGGTACTCGGTCATCCAGGTG		
BAX	F: TCCATTCAGGTTCTCTTGACC	XM_001235092.4	119
	R: GCCAAACATCCAAACACAGA		
Caspase-3	F: TACCGGACTGTCATCTCGTTCAGG	XM_015276122.2	166
	R: ACTGCTTCGCTTGCTGTGATCTTC		
ZO-1	F: ACACTGTGACCCCAAAACCT	XM_015278981.2	81
	R: ACTGAGACACAGTTTGCTCCA		
Occludin	F: CATCGCCTCCATCGTCTACA	NM_205128.1	117
	R: TTGAGGTAGGTGCTGCCGTA		
β-actin	F: GTGCTATGTTGCTCTAGACTTCG	NM_007393.5	174
	R: ATGCCACAGGATTCCATACC		

## Data Availability

Not applicable.

## Sample Availability

Samples of the compounds are not available from the authors.
